# Neurodegeneration at the crossroads: the gut-brain axis and blood-brain barrier in Parkinson’s disease — a review

**DOI:** 10.3389/fphar.2026.1813134

**Published:** 2026-05-08

**Authors:** Nada K. Gamal, Rafik Fakhry, Youmna Hatem, Engy Rashed, Reem Marzouk, Ahmed K. M. Bukr, Nadia Akawi, Mina Y. George

**Affiliations:** 1 Department of Pharmacology and Toxicology, Faculty of Pharmacy, Ain Shams University, Cairo, Egypt; 2 PharmD Clinical Program, Faculty of Pharmacy, Ain Shams University, Cairo, Egypt; 3 Department of Genetics and Genomics, College of Medicine and Health Sciences, United Arab Emirates University, Al Ain, United Arab Emirates; 4 Biology Department, School of Pharmacy, Newgiza University, Giza, Egypt

**Keywords:** blood-brain barrier, gut microbiota, gut-brain axis, inflammation, neuroprotection, Parkinson’s disease

## Abstract

Parkinson’s disease (PD), which is one of the most common neurodegenerative illnesses, involves abnormal deposition of α-Synuclein and loss of dopaminergic neurons in the substantia nigra. Beyond this, there is increasing evidence that the gut-brain axis (GBA) and blood-brain barrier (BBB) interfere in disease initiation and progression. Dysbiosis of the gut microbiota affects the intestine and the BBB, allowing microbial metabolites and proinflammatory mediators to enter the CNS, causing neuroinflammation and neurodegeneration. Studies show that α-Synuclein pathology can originate in the gut and reach the brain via the vagus nerve. This review summarizes the connections among GBA, BBB, and PD, focusing on oxidative damage, inflammatory cascades, decreased expression of tight junction proteins, and signaling pathways such as TLR4/MyD88/NF-κB. In addition, we discuss therapeutic strategies that target the microbiota-BBB axis, such as probiotics, fecal microbiota transplantation, natural compounds (e.g., piperine, anethole, polymannuronic acid, Paeonia lactiflora), and stem cell therapy, which have demonstrated neuroprotective potential in animal models. Overall, the literature emphasizes the importance of restoring gut homeostasis and BBB integrity, and suggests that getting this axis right may offer novel opportunities for PD treatment. Future research is crucial to validate the efficacy of this approach clinically and to develop tailored therapies to prevent or delay PD progression.

## 1 Introduction

Parkinson’s disease (PD), a prevalent neurodegenerative disease, is a complex condition that drastically impacts the quality of a patient’s life through motor manifestations, including rigidity, postural instability, resting tremor, and bradykinesia, leading to impaired balance and mobility in addition to other non-motor manifestations like sleep problems, gastrointestinal disturbances, anxiety, and depression (Chaudhuri et al., 2006; Kalia and Lang, 2015; Moustafa et al., 2016; Rong et al., 2021; Desouky et al., 2023). The pathological hallmark of PD is the gradual loss of dopaminergic neurons in the substantia nigra (SN) due to the accumulation of Lewy bodies, which are formed by aberrant aggregates of α-Synuclein (α-Syn) protein. This monomeric protein has elevated β-sheet content and relatively low α-helical content, and it misfolds readily following transcription. Eosinophilic intracellular deposits accumulate due to this misfolding, forming insoluble amyloid fibrils (Tofaris, 2022; Mohammed et al., 2024). Although most commonly found in the brain, α-Syn aggregates have also been identified in peripheral tissues, especially in the enteric nervous system, supporting the idea that the CNS is not the only system involved in PD, but that peripheral systems are also implicated (Ding et al., 2018; Chao et al., 2020).

The blood-brain barrier (BBB) is a semipermeable membrane that is found at the interface between systemic circulation and the CNS, selectively controls the exchange of molecules, Ions, and signals from the blood to the brain, and ensures the stability required for neuronal function (Beltran-Velasco and Clemente-Suárez, 2025). It consists of tight junctions, efflux transporters, and metabolic enzymes that prevent the entry of most xenobiotics (Yang, 2025). Its main role is to regulate the flow of molecules, cells, and signals between these compartments. Nonetheless, its disruption has been related to neurodegenerative illnesses like PD (Beltran-Velasco and Clemente-Suárez, 2025).

Alongside α-Syn propagation, there is an increased focus on the interplay between the gut microbiota composition, intestinal barrier integrity, and PD. Recently, the interplay between PD and gut microbiota composition has attracted significant interest in the study of neurodegenerative disorders. The gut microbiota is the diverse bacterial, fungal, and viral population living in the intestine, forming the most sophisticated microbial ecosystem in the human body. It comprises approximately 50 bacterial phyla, with Bacteroidetes and Firmicutes accounting for more than 90% of the overall population (Ding et al., 2021; Guo et al., 2025). The alterations in this composition lead to increased mucosal immune activation, affecting the intestinal epithelium’s integrity and permeability, permitting cytokines and endotoxins to reach the systemic circulation. These circulating mediators have the ability to activate inflammatory pathways, disrupt the BBB, and promote neuroimmune crosstalk (Parker et al., 2020). Microglial stimulation and oxidative stress cause a feedback loop that increases α-Syn aggregation and dopaminergic neuron death (Shenthilvel et al., 2026). Recent research suggests that gut microbial dysbiosis is a clinical indication of Parkinson’s disease development. Cumulative evidence validates that gut dysbiosis could be a clinical indicator feature of PD progression, potentially affecting α-Syn aggregation and neuroinflammation (Lubomski et al., 2020; Yadav and Raj, 2025). These findings have increased interest in microbiome-targeted therapeutics and established the gut as a new and promising pathway for disease modulation. A better understanding of these intricate host-microbiota interactions is important, since it may lead to new treatments and enhance the therapeutic landscape of PD.

## 2 Parkinson’s disease beyond neurons

The accumulation of misfolded α-Syn and the progressive loss of nigrostriatal dopaminergic neurons have been extensively utilised to define PD as a neurodegenerative motor disease (Poewe et al., 2017). Nevertheless, growing data indicates that PD encompasses different system dysfunctions, in addition to central neurodegeneration, particularly at physiological interfaces that regulate interactions between the central and peripheral systems.

Gastrointestinal symptoms are the most frequently reported non-motor symptoms, including constipation, dysphagia, and bloating, which appear years before motor symptoms (Chiang and Lin, 2025). Such early prodromal manifestations suggest that PD may originate outside of the brain, with central neurodegeneration occurring as a consequence of additional systemic physiological alterations. Moreover, Investigations have shown α-Syn accumulation in the enteric nervous systems of PD patients, before evident central pathology (Chao et al., 2020).

In addition to CNS pathology, changes in the gut microbial ecology have been observed in several datasets of PD patients. Furthermore, Sequencing studies revealed a decrease in short-chain fatty acid (SCFA)-producing bacteria and an increase in pro-inflammatory Gram-negative bacteria that produce the endotoxin, lipopolysaccharide (LPS) (Keshavarzian et al., 2015; Scheperjans et al., 2015). This microbial dysbiosis in PD patients may contribute to enhanced pro-inflammatory mediators, intestinal permeability, and mucosal immune activation, suggesting that microbial dysbiosis may also contribute to a systemic inflammatory environment (Shenthilvel et al., 2026).

At the same time, clinical investigations have reported enhanced intestinal permeability in PD, which is known as “leaky gut,” wherein lactulose/mannitol ratios are higher than in healthy controls (Forsyth et al., 2011). Colonic biopsies from patients have demonstrated reduced expression of tight junction proteins such as occludin and zonula occludens-1 (ZO-1), supporting degradation of the epithelial barrier (Forsyth et al., 2011).

Besides the intestinal barrier disruption in PD, the BBB, which shields the brain from its surroundings, is disrupted early in PD. Studies indicated increased BBB permeability and decreased tight junction proteins (Lau et al., 2024). When the barrier is disrupted, inflammatory cytokines, endotoxins, and misfolded proteins can translocate to the brain, causing neuroinflammation and microglial activation, which in turn deteriorates BBB integrity and dopaminergic neuron susceptibility (Lau et al., 2024; Shenthilvel et al., 2026).

These findings support a barrier-centric view of PD that emphasizes that early disruption of the intestinal epithelium and BBB may cause or worsen neurodegeneration and that the potential of treatment approaches that maintain both barrier integrity and neuronal health. Early disruption of the gut epithelium and BBB may cause or worsen neurodegeneration.

## 3 The cross-talk between gut microbiota and the intestinal barrier

The microbiota is a group of symbiotic and commensal microorganisms that has a density of more than 10^12^ cells per Gram of the large intestine in humans. A well-balanced relationship between the microbiota and the host is crucial for both intestinal health and the body as a whole (Kim et al., 2017). Interestingly, microbiota develops and stabilizes by weeks three to four in mice and by the ages of two to 3 years in humans. To shape immune tolerance to commensal microbes and maintain mucosal integrity, microbiota first interact with mucosal site immunity and preserve gut barrier integrity (Tierney and Nelson, 2009; Schloss et al., 2012; Stecher, 2015). Concurrently, the distal organs are affected, including the brain, which is particularly involved in neuronal development and is supported and shaped by maternal microbiota (Tierney and Nelson, 2009; De Palma et al., 2015; Gomez de Agüero et al., 2016; O’Mahony et al., 2017; Gamal et al., 2025). The microbiota is extremely complex, and its composition can change, especially after switching from lactation to solid foods. It is constantly influenced by a wide range of internal variables, including intestinal pH, temperature, intestinal secretions, bile acids, and immune responses, and other external factors, including diet and environment. Trillions of germs, including over 40,000 species of bacteria, make up the gastrointestinal tract’s large and diverse microbial environment in adulthood (Nishino et al., 2013). Interestingly, gut microbiota interactions play a key role in maintaining a person’s immunity, as the gut accounts for a significant portion of the immune system. It promotes acquired immunity by boosting both systemic and local immunological reactions and the metabolism of specific nutrients. It also activates the innate immune system early in development, thereby promoting the maturation of gastrointestinal-associated lymphoid tissue (D’Amelio and Sassi, 2018; Logsdon et al., 2018). Moreover, the microbiota produces SCFAs, such as butyrate, propionate, and acetate, that have a role in maintaining the intestinal mucosa and barrier. It has also been demonstrated that SCFAs enhance the efficiency and quantity of gut-resident regulatory T cells (Tregs), supporting immunological homeostasis (Braniste et al., 2014; Akhtar et al., 2022). The intestinal barrier is composed of a layer of epithelial cells connected by tight junctions, mucus, and immunological constituents. It blocks bacteria, poisons, and pro-inflammatory substances from entering the circulation while permitting selective absorption of nutrients (Chelakkot et al., 2018). Homeostasis of this ecosystem is crucial, since its disruption by diet, drugs, and aging potentially promotes gut inflammation, dysbiosis, and immune cells and pro-inflammatory factors, compromising the intestinal barrier, resulting in enhanced intestinal permeability, evident by decreased expression of tight junction proteins (occludin, ZO-1) and changed epithelial shape. This increased permeability leads to translocation of endotoxins and proinflammatory substances to the systemic circulation (Clairembault et al., 2015; Kelly et al., 2015; Mou et al., 2022).

Consequently, this leads to sustained systemic inflammation, which results in remodeling of the BBB with increased permeability and collapse, activating microglia in the brain and stimulating both neuroinflammation and neurodegeneration (Kebir et al., 2007; Huppert et al., 2010; Rahman et al., 2018; Mou et al., 2022). BBB is already considered a hallmark of neurological illness; its breakdown can be immune-mediated, its aging begins in middle age in both humans and rodents, and its collapse can serve as a biomarker for cognitive dysfunction, alongside both amyloid and tau (Nation et al., 2019; George et al., 2023; Aboelnasr et al., 2025). One important structural component that limits paracellular transport and preserves the low permeability needed to protect the brain is the BBB junctional complex. Tight junctions are an essential component of the BBB, as they restrict the entry of both endogenous and exogenous substances into the brain. Disruption of tight junctions significantly increases BBB permeability, leading to neuroinflammation and neurological disorders (Abou Diwan et al., 2023).

Emerging studies suggest a close association between the gut microbiota and BBB integrity. Experimental studies have shown that bacterial-derived components, such as lipopolysaccharide (LPS) and lipoteichoic acid (LTA), can directly or indirectly, through immune activation, lead to BBB dysfunction. Germ-free mice showed reduced expression of tight junction proteins and impaired BBB integrity. However, reintroducing normal flora restores these functions, suggesting that some commensal bacteria and/or their metabolites resolve BBB dysfunction (Boveri et al., 2006; Salkeni et al., 2009; Banks et al., 2015; Logsdon et al., 2018).

Dietary components, including fibers, polyphenols, and macro- and micronutrients, are transformed by the gut microbiota into a range of metabolites, including vitamins, triethylamines, SCFAs, and amino acid derivatives. They have essential metabolic and signaling functions that can modulate host homeostasis, including both BBB integrity and brain function (Parker et al., 2020). SCFAs, like acetate, propionate, and butyrate, are particularly crucial in promoting tight junction protein production and decreasing BBB permeability. A study showed that restoring SCFAs in germ-free mice upregulated the expression of tight junction proteins, such as occludin and claudin-5, thereby reducing BBB permeability. In contrast, the absence of microbial metabolites was associated with aberrant tight junction disruption, increased susceptibility to systemic inflammation, and enhanced barrier leakage (Braniste et al., 2014). Metabolite-immune cell interactions in the gut may reach the brain by various ways, like the release of cytokines and other immune signaling molecules into the bloodstream, which eventually reach the BBB and when the epithelial barrier is broken down because of dysbiosis, damage, infection, or age-related degeneration, eventually, bacteria, their metabolites, and other substances like toxins and LPS can enter and cause damage to enteric neurons as well as inflammatory immune responses. Maintaining a vicious cycle of neurodegeneration and inflammation in the brain is associated with multiple neurological and neurodegenerative diseases (Braniste et al., 2014; Logsdon et al., 2018; Parker et al., 2020; Ibrahim et al., 2021).

In the hypothesis that the gut microbiome plays a major role in disrupting the BBB in spontaneously hypertensive stroke-prone rats (SHRSP), altering the gut microbiome was more prevalent in the SHSPR than in the control group. Inflammation results from changes in the gut microbiome. Gut inflammation and interleukin (IL)-17 were upregulated, potentially involving endothelial dysfunction and cognitive impairment. These issues can cause hypertension at a young age (George et al., 2026). Moreover, the SHRSP microbiome would tend to inflame the gut and could translocate bacterial toxins and bacteria. The gut was the initial site for inflammation. It may eventually become systemic and reach the brain. Due to gut dysbiosis, Proinflammatory T cells migrate to the brain following stroke, thereby initiating neuroinflammation that causes cerebral small vessel disease. While hypertension worsens disease severity, BBB disruption occurred regardless of systolic blood pressure, implying that microbiome-driven inflammation is a critical factor (Nelson et al., 2021).

**FIGURE 1:**
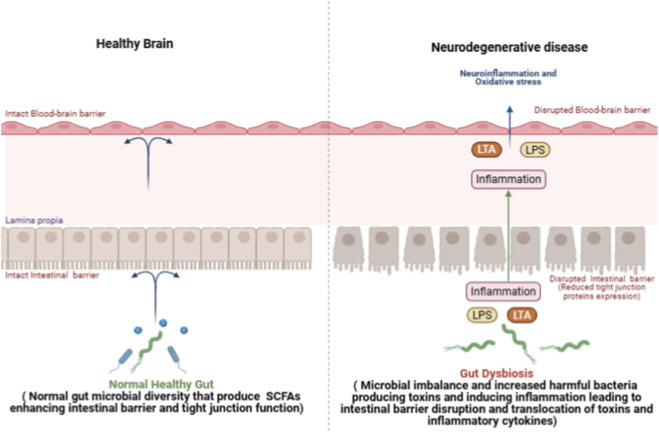
The crosstalk between gut dysbiosis and Blood-Brain barrier integrity in health and neurodegenerative diseases. In the healthy state (left panel), both the blood–brain barrier (BBB) and intestinal epithelial barrier remain structurally intact, supported by tight junction integrity. A balanced gut microbiota produces beneficial metabolites such as short-chain fatty acids (SCFAs), which enhance epithelial barrier function, maintain immune homeostasis, and support normal brain physiology. In neurodegenerative conditions (right panel), gut dysbiosis characterized by microbial imbalance and increased pathogenic bacteria leads to elevated production of lipopolysaccharide (LPS) and lipoteichoic acid (LTA). These microbial products promote intestinal inflammation, reduce tight junction protein expression, and disrupt the intestinal barrier, allowing translocation of toxins and pro-inflammatory cytokines into the circulation. The resulting systemic inflammation contributes to BBB disruption, facilitating neuroinflammation and oxidative stress within the central nervous system (CNS), ultimately promoting neurodegenerative processes (Created in https://BioRender.com).

Additionally, disruption of the gut microbiota has been demonstrated to worsen ischemic stroke by compromising the intestine and BBB integrity via inflammation-induced pathways. In a middle cerebral artery occlusion/reperfusion model, gut dysbiosis was associated with elevated pathogenic taxa, such as Escherichia-Shigella, and increased circulating LPS levels. This activated the Toll-like receptor 4 (TLR4)/myeloid differentiation primary response 88 (MyD88)/nuclear factor kappa B (NF-κB) inflammatory signaling pathway, leading to systemic and neuroinflammation (Hanna et al., 2023). A common component of Panax notoginseng, notoginsenoside R1 (NG R1), improved gut microbiota balance, decreased LPS translocation, and increased tight junction protein expression in both the gut and brain barriers. These effects together maintained BBB integrity, hindered glial stimulation, and reduced ischemia injury. Furthermore, fecal microbiota transplantation from NG-R1-treated rats recapitulated these protective effects, underscoring the critical role of the microbiota-gut-brain axis (Zhang et al., 2024). The link between the microbiota and the BBB is summarized in Figure 1.

## 4 The blood-brain barrier and Parkinson's disease

**FIGURE 2:**
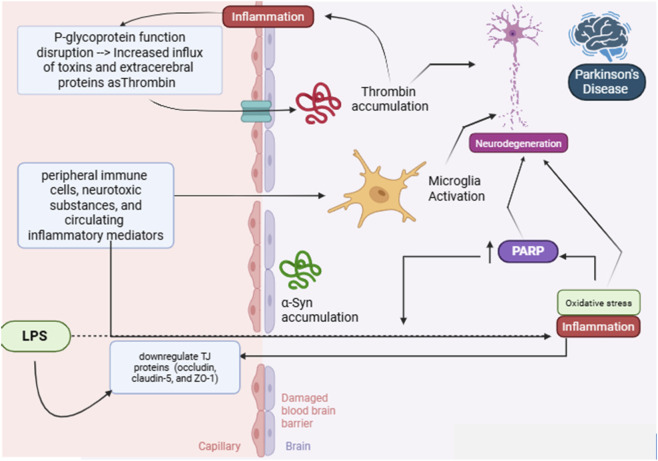
The role of the Gut-brain axis in Parkinson’s disease through the modulation of neuroinflammation, alpha-synuclein, tight junction proteins, oxidative stress, and neurodegeneration. Schematic representation of the molecular and cellular mechanisms linking inflammation, blood-brain barrier (BBB) disruption, and neurodegeneration in Parkinson’s Disease. Lipopolysaccharide (LPS) exposure leads to downregulation of tight junction (TJ) proteins (occludin, claudin-5, and ZO-1), resulting in a damaged BBB. This disruption allows increased influx of peripheral immune cells, neurotoxic substances, and circulating inflammatory mediators into the brain. Concurrently, P-glycoprotein dysfunction exacerbates the accumulation of toxins and extracerebral proteins such as thrombin within the brain. Thrombin accumulation and α-synuclein (α-Syn) aggregation trigger microglial activation, leading to neuroinflammation and oxidative stress. These processes promote poly (ADP-ribose) polymerase (PARP) activation, which contributes to neurodegeneration and the progression of Parkinson’s Disease (Created in https://BioRender.com).

There is growing evidence that the BBB actively contributes to the development and progression of disease, rather than just acting as a passive barrier, and acts as the main line of defense against PD (Lee, 2014) (Figure 2). In healthy conditions, the BBB consists of specialized cerebral endothelial cells attached by tight junction proteins that regulate paracellular permeability and maintain the homeostasis of the CNS. ZO-1 and claudin-5 are considered the main tight junction proteins. ZO-1 links the transmembrane to the actin cytoskeleton, which helps to maintain the structure of endothelial cells. Claudin-5 works as the main component that seals the junctions, controlling the diffusion of small molecules. In addition to the integrity of the tight junction, for optimal BBB function, an organized interaction between the neurovascular unit, including pericytes, astrocytic end-feet, basement membrane proteins, and microglial supervision, is also required to preserve selective transport, low permeability, and vascular signaling necessary for neuronal homeostasis (Lau et al., 2024).

The disruption of the BBB integrity enhances microglial activation. It promotes dopaminergic neuronal death in the SN by facilitating the invasion of peripheral immune cells, neurotoxic substances, and circulating mediators of inflammation (Lee, 2014; Guo et al., 2025). The increase in BBB permeability in the SN in PD promotes α-Syn aggregation and dopaminergic cell death, worsening PD symptoms (Beltran-Velasco and Clemente-Suárez, 2025). PD symptoms are motor symptoms (resting tremors, muscle rigidity, bradykinesia, akinesia, and postural instability) and non-motor symptoms (anxiety, depression, restless leg syndrome, fatigue, hypotension, and bladder and bowel problems). Those symptoms often develop and worsen as the BBB gets disrupted due to inflammation, oxidative stress, pathological conditions, genetic enzymatic expressions, neuronal firing and accumulation of extracerebral proteins which often occur due to agricultural pesticides like rotenone, a neurotoxin which induces PD by increasing BBB permeation proved using Evans blue dye, rotenone causes increased activity of microglia and matrix metalloproteinases (MMP-2 and MMP-9) leading to reduced tight junction proteins, ZO-1, claudins and occludins expression and thus a damaged-leaky BBB which resulted in the accumulation of fibrinogen in the hippocampus. Interestingly, PLX3397 and minocycline both reduced rotenone-induced microglia activity, decreased Evans blue permeation, restored tight junction protein expression, and hindered MMP2-9. Likewise, SB-3CT, the MMP-2/-9 inhibitor, safeguarded the BBB from rotenone-induced damage, demonstrating a neuroprotective effect. This was achieved by restoring the BBB integrity through enhancing ZO-1 and claudin expression. As a result, the expression of the pro-apoptotic protein Bax and active caspase-3 decreased, alongside elevated anti-apoptotic Bcl-XL levels. SB-3CT also showed increased neuronal nuclear antigen (NeuN) immunoreactivity, indicating the preservation of neuronal structural integrity, and also restored postsynaptic density protein-95 (PSD-95), a key postsynaptic scaffolding protein for synaptic integrity (Morid et al., 2023). These findings supported that the BBB stabilization can halt both inflammatory and apoptotic neurodegeneration processes in PD (Guo et al., 2022).

Recent research has demonstrated that, by maintaining BBB integrity, anethole, a naturally occurring substance with strong antioxidant activity, exerts neuroprotective effects in rotenone-induced PD mice. Anethol’s antioxidant effect prevented α-Syn and monoamine oxidase-B accumulation, reestablished striatal neuronal firing activity, and improved coordination and neuronal function, as demonstrated by tests such as the rotarod test, stride length test, and motor coordination tests, suggesting a neuroprotective property of anethol and fennel constituents (Moradi Vastegani et al., 2023).

PD symptoms worsen due to inflammation which may occur due the presence of specific factors like multidrug resistance protein 1 (MDR1), causing genetic expression of MDR1 which disrupts P-glycoprotein in BBB allowing passage of extracerebral proteins like thrombins that accumulate in SN causing direct inflammation and toxicity of cells in SN and striatum which led to the use of P-glycoprotein modulators to protect the BBB integrity, those modulators include progesterone, heat shock protein 90 and novel nano-pharmaceutical technology and σ2 receptor antagonists to avoid expressing MDR1 a predisposing gene to PD (Lee, 2014). In addition, using microdialysis, BBB permeation was tested, proving that inhibiting P-glycoprotein by zosuquidar increases BBB permeability, which was significantly decreased when treated with FLZ, a highly plasma protein-bound drug of 90% binding, up to fourfold when compared to FLZ treatment without zosuquidar, and free FLZ AUC was 3-fold higher in FLZ combined with zosuquidar treatment than FLZ alone, which indicates that zosuquidar enhanced permeation of FLZ. Inflammation may also be a consequence of the pathological condition Lewy body disease, which precedes PD, causing increased BBB permeation when tested with immunofluorescent stains, showing extravascular erythrocytes and hemosiderin deposits, and eosin and hematoxylin in the striatal BBB, where PD brains showed 7 times more extravasated erythrocytes in the striatum compared to normal brains (Hou et al., 2014). In PD, poly (ADP-ribose) polymerase (PARP), an enzyme activated by inflammation and oxidative stress, plays a significant role in disrupting the BBB by downregulating tight junction proteins, including occludin, claudin-5, and ZO-1. Overactivation of PARP has been demonstrated to harm dopaminergic neurons and jeopardize the integrity of the BBB. In experimental animals, LPS exposure led to dopaminergic neuronal damage and increased BBB permeability by inhibiting tight junction proteins. Administration of 3-aminobenzamide dramatically reduced BBB permeability and protected dopaminergic neuron survival, principally by upregulating ZO-1, occludin, and claudin-5 expression. The results presented suggest that PARP inhibition may protect against neuroinflammation-induced disruption of the BBB and dopaminergic degeneration, making it a promising treatment option for PD (Wu et al., 2014).

## 5 Parkinson's disease and gut-brain axis

Although dopamine replacement medications continue to be the primary treatment for early motor manifestations, developing cytoprotective therapies to prevent the formation of Lewy bodies has remained challenging. Moreover, Research shows that BBB integrity and GBA interact in PD. Levodopa is the primary symptomatic therapy for PD; nevertheless, accumulating data suggest bidirectional interactions between levodopa and the gut microbiome. Several bacterial taxa have decarboxylase activity, allowing them to metabolize levodopa in the intestine before its absorption, lowering its bioavailability and contributing to interindividual variability in treatment response. Its chronic administration has been linked to compositional changes in the gut microbiome, including enrichment of taxa such as *Lactobacillus* and *Enterococcus* species, both repeatedly reported in PD microbiome studies, demonstrating that long-term dopaminergic therapy may partially modify microbial metabolic networks (van Kessel et al., 2019). This could lead to dysbiosis, which can be associated with increased intestinal permeability and systemic inflammation, which may compromise BBB integrity and enhance neuroinflammatory processes along GBA. Subthalamic nucleus deep brain stimulation (STN-DBS), although mainly targeting motor circuitry, has been linked to the regulation of specific dysbiotic microbiota patterns and the systemic inflammatory mediators, indicating a secondary role in maintaining GBA balance (Gorecka-Mazur et al., 2024).

Piperine was demonstrated to be a potential modulator by restoring microbiota balance, improving intestinal barrier function, and reducing dopaminergic neuronal death. Piperine administration alleviated both gastrointestinal and motor impairment in PD models. Piperine inhibited the phosphoinositide 3 kinase (PI3K)/Akt/mammalian target of rapamycin (mTOR) pathway, inducing autophagy in the colon and SN and reducing α-Syn deposition. Piperine repaired dysbiosis by reducing harmful bacteria such as *Escherichia coli* and *Salmonella* while increasing *Prevotella*, which produces SCFAs that, in turn, help maintain the intestinal barrier and BBB integrity. Moreover, piperine reduced peripheral inflammation and microbial amyloid-induced α-Syn aggregation, indirectly protecting the BBB and restricting systemic immune activation and neuroinflammation (Yu et al., 2024). Interestingly, the stem cell therapy approach aims to restore dopaminergic function by replacing lost neurons with Embryonic stem cells, Induced pluripotent stem cells, and Mesenchymal stem cells. It was found that intra-gut stem cell transplantation worsened PD motor and non-motor symptoms, coupled with upregulation of inflammation-relevant microbiota and cytokines in the gut and brain of transgenic mice, because it alters the colonic barrier, allowing the passage of bacterial metabolites into the blood and worsening motor symptoms. Moreover, PD patients exhibit a leaky gut, and stem cell transplantation can even worsen this condition, thereby impairing motor performance, as the gut is already inflamed during the progression of PD pathology. A traumatic intervention, such as the present transplantation into the superior mesenteric artery, likely compounded the gut’s inflammatory response to the disease progression. The three microbiota that are identified as associated with gut inflammation, namely, BAC303, EREC482, and LAB158, were all significantly elevated in transplanted transgenic mice, leading to upregulation of inflammatory cytokines, including Tumor necrosis factor-alpha (TNF-α), ultimately leading to accumulation of α-Syn in both gut and brain. The intravenous transplantation of stem cells reduced gut-inflammation-associated microbiota and cytokines, coinciding with the rescue of behavioral and histological deficits in PD animal models. Also, the transplanted stem cells may activate the GBA signaling pathway by isolating gut-derived inflammatory signals, thereby dampening the inflammation-mediated neurodegenerative cascade of cell death in the brain (Lee et al., 2023).

Growing evidence of a bidirectional relationship between the gut and the brain suggests that probiotics may be used to improve gut microbiota health and stability, thereby potentially preventing or delaying neurodegenerative diseases. Probiotic or prebiotic supplementation has been shown to increase glycolysis and mitochondrial activity, potentially enhancing energy metabolism in both muscle and brain in a rat model of PD. Consequently, these effects may help prevent muscle atrophy and the loss of dopaminergic neurons. Supplementation also modulated SCFA production and increased antioxidant enzyme activity, thereby protecting mitochondria from ROS-induced damage. Probiotics can further restructure the composition of gut microbiota, enhancing the host’s anti-inflammatory and antioxidant capacity. For example, supplementation with *Lactobacillus salivarius AP-32* altered gut microbiota composition in PD-induced rats, increasing beneficial bacteria, including *Lactobacillus*, *Eggerthella*, *Prevotella*, and *Megamonas*, while suppressing pathogenic taxa, such as *Clostridium* and *Ruminococcaceae*. Mechanistically, probiotics may act by stimulating host antioxidant defenses, increasing SCFA production, particularly butyrate and propionate, which have potent anti-inflammatory properties, reducing harmful byproducts such as hydrogen peroxide via enhanced microbial catalase and superoxide dismutase activity, and improving intestinal barrier integrity. Collectively, these effects reduce systemic inflammation and prevent harmful leakage across the BBB, thereby mitigating the pathological aggregation of α-Syn (Tsao et al., 2021). Among potential probiotic interventions, the genus *Bifidobacterium* has been extensively studied for its health-promoting effects in neurodegenerative disorders, including PD. Specific strains such as *Bifidobacterium breve CCFM1067* have shown protective effects in the 1-methyl-4-phenyl-1,2,3,6-tetrahydropyridine (MPTP) model of PD, where they restored microbiota composition and preserved gut barrier integrity. Moreover, *Bifidobacterium* supplementation has been reported to exert anti-inflammatory effects by directly regulating the expression of proinflammatory cytokines in macrophages. This is particularly relevant to PD, where elevated cytokine secretion and activation of inflammatory transcription factors in neurons contribute to disease progression. In addition to its immunomodulatory properties, *Bifidobacterium* may influence dopaminergic signaling. Tyrosine hydroxylase (TH), the rate-limiting enzyme in dopamine biosynthesis, serves as an established marker of PD progression. Studies have shown that TH levels decline in both PD patients and animal models, reflecting dopaminergic neuron loss. Interestingly, treatment with higher doses of *Bifidobacterium breve Bif11* restored TH expression in the midbrain, suggesting a compensatory effect on dopamine biosynthesis and signaling. Collectively, these findings highlight the therapeutic promise of *Bifidobacterium* in modulating gut microbiota, reducing inflammation, and supporting dopaminergic function in PD (Valvaikar et al., 2024).

Recent research indicates that modification of the gut microbiota may underlie the therapeutic effects of traditional medicines in PD. Er-Bai-Tang decoction enhanced the intestinal flora, which directly correlated to PD. In PD, p38 mitogen-activated protein kinases (MAPK) are activated in dopaminergic neurons in the SN, leading to dopaminergic neuronal loss in patients. Inhibition of MAPK activation has recently been shown to be a key PD treatment pathway. When the EBT decoction was administered, it reversed motor impairment in PD model rats and reduced cell necrosis in the SNpc.

Furthermore, the EBT decoction inhibited the exaggerated expression of p38 MAPK mRNA and protein in PD model rats. Upon investigation, the intestinal flora composition was examined at three distinct levels: phylum, family, and genus. At the family level, Rikenellaceae was the key species and showed a marked increase in the PD model groups. At the genus level, *Alistipes* and *Allobaculum* also increased significantly in model rats. These results suggested that EBT could improve the composition of the gut microbiome. Compared with control rats, PD model rats also showed a drastic increase in Sphingolipid metabolism in their intestinal flora. EBT treatment mainly inhibited the biosynthesis of siderophore group non-ribosomal peptides in the intestinal flora of the model rats (Li et al., 2022). *Paeonia lactiflora Pall*., a popular Chinese medicinal plant, has shown significant neuroprotective effects in an MPTP-induced mouse model of PD. *Paeonia lactiflora* water extract treatment prevented BBB disruption and neuroinflammation, decreased dopaminergic neuronal death in the SN and striatum, and markedly improved motor deficits. Physiologically, these positive effects were linked to gut microbiota remodeling, namely, a higher abundance of the genus *Dubosiella*, as determined by 16S rRNA sequencing and metabolomic profiling. Its neuroprotective effects seemed to be mediated by the formation of serum indoleacetic acid, a metabolite with neuroprotective and anti-inflammatory properties. These effects seemed to be mediated by the formation of serum indoleacetic acid, a metabolite with neuroprotective and anti-inflammatory properties. These results demonstrate the therapeutic potential of microbiota-derived metabolites and Paeonia lactiflora in the treatment of PD by preserving BBB integrity and dopaminergic cell survival through modulation of the gut–brain axis (Shao et al., 2025).

Furthermore, another study examines how the gut microbiota and associated metabolic pathways contribute to rotenone-induced neurotoxicity. The data showed that in rotenone-induced PD mice, gut dysbiosis is associated with lower levels of SCFA, particularly acetate and butyrate, which ordinarily sustain intestine and BBB integrity. When these metabolites are lost, barrier function is compromised, and systemic inflammation is triggered by elevated TNF-α and IL-6 levels. Furthermore, there was a significant drop in nicotinamide adenine dinucleotide (NAD+), a consequence of the nicotinate/nicotinamide pathways. Solute Carrier Family 25 Member 51 and nicotinamide phosphoribosyl transferase levels have also decreased. After analyzing these findings, it was concluded that rotenone exposure directly affected the gut microbiota. The intestine’s permeability changed, and inflammatory responses were triggered when the levels of butyric and acetic acids decreased. The study found that although gut microbiome diversity did not differ significantly, the rotenone-exposed group showed substantial alterations in gut microbiome species compared with the control group. A reduction in *Lactococci, Bacteroidetes, and Prevotellaceae* demonstrated this. *Firmicutes’* relative abundance has also grown. Two gut microorganisms were of interest: *Bacteroidetes*, a fundamental member of the gut microbiota, plays an important role in maintaining the gut barrier’s stability and integrity. Then comes *Prevotellaceae*, which is important for intestinal mucin synthesis. When its quantity declines, the intestinal barrier becomes more porous, increasing exposure to bacterial endotoxins. This triggers an intestinal inflammatory response, leading to the overproduction of α-Syn protein in the intestine. This directly supports the PD-GBA correlation (Sai et al., 2025).

A recent study showed that by restoring the gut ecosystem and strengthening BBB integrity through the AMP-activated protein kinase/superoxide dismutase 2 (AMPK/SOD2) signaling pathway, fecal microbiota transplantation (FMT) from healthy human donors reduces neurodegeneration in a PD mouse model induced by MPTP. In this study, FMT from PD patients exacerbated intestinal inflammation, disrupted the AMPK/SOD2 axis, promoted glial activation, caused dopaminergic neuron loss, and induced motor deficits. Remarkably, FMT from healthy individuals reversed these consequences, restoring gut homeostasis, reducing microgliosis, maintaining tyrosine hydroxylase-positive neurons in the SN, and improving behavioral impairments (Xie et al., 2023). Building on these results, another study has further emphasized the therapeutic benefits of FMT by directly restoring gut microbiota balance in PD. Fecal samples from rotenone-induced mice showed increased levels of the bacterial genera *Akkermansia* and *Desulfovibrio*. Healthy FMT restored gut dysbiosis, enhanced gastrointestinal function, and significantly improved motor function in a rotenone-induced mouse model by reducing systemic LPS leakage when lowering intestinal inflammation and maintaining the integrity of the barrier. Thus, neuroinflammation was reduced, and dopaminergic neurons were preserved by suppressing LPS–TLR4/MyD88/NF-κB pathway activation in the gut and the SN. Interestingly, healthy FMT reduced BBB disruption and thereby prevented inflammatory cytokines from penetrating the CNS (Zhao et al., 2021). Furthermore, recent research revealed that panaxadiol, a ginseng-derived bioactive sapogenin, ameliorates motor and gastrointestinal impairment in rotenone-induced PD rats while preserving dopaminergic neuronal integrity. dopaminergic neuronal integrity. Notably, panaxadiol improved gut microbial dysbiosis by counteracting rotenone-induced changes in microbial diversity and abundance, and restored BBB integrity by enhancing the expression of tight junction proteins, such as ZO-1, occludin, and claudin-5, thereby restricting the infiltration of peripheral inflammatory mediators into the SN. panaxadiol suppressed inflammation centrally and peripherally by inhibiting the TLR4/MyD88/NF-κB signaling pathway (Xu et al., 2025).

A recent study showed that polymannuronic acid, a polysaccharide from brown seaweed, could be a potential microbiota-targeted treatment for PD. In an MPTP-induced mouse model of PD, administration of polymannuronic acid restored motor function. It preserved dopaminergic neurons in the SN by reducing systemic, gut, and neuroinflammation, as demonstrated by reduced proinflammatory cytokine production and reduced MAPK signaling in the colon. Moreover, Polymannuronic acid enhanced intestinal and BBB integrity, as proved by increased tight junction protein expression in the colon and SN. Furthermore, gut microbiome profiling showed that polymannuronic acid altered the microbiota and increased SCFAs levels, indicating that microbiota-derived metabolites have an important role in its neuroprotective effects (Dong et al., 2020). In addition to natural substances studied, an investigation has shown that coffee may have a neuroprotective effect in MPTP-induced PD mice. It has been demonstrated that coffee administration significantly reduced α-Syn accumulation, attenuated dopaminergic neuronal degeneration, and improved motor function by enhancing the tight junction protein occludin and thereby BBB integrity, while suppressing the marker of astrocyte activity and neuroinflammation, glial fibrillary acidic protein.

Furthermore, coffee inhibited pro-apoptotic signaling by decreasing Bax, cleaved caspase-3, and cytochrome c levels while boosting the anti-apoptotic protein Bcl-2, preventing neuronal apoptosis. Remarkably, coffee reduced MPTP-induced gut microbiota dysbiosis, suggesting that its benefits extend beyond central neuroprotection to modulation of the gut-BBB-brain axis (Liu et al., 2022). This shows that targeting the GBA with nutritional components, microbiota-derived metabolites, or microbiota-regulating medications could be an effective complementary strategy for maintaining the BBB and halting PD progression, as represented in Table 1.

**TABLE 1:** Studies targeting the gut-brain axis to maintain blood-brain barrier integrity in Parkinson’s disease.

Suggested treatment/Experimental model	Dosage	Key findings	References
Piperine (6-hydroxydopamine (6-OHDA)-lesioned male Sprague-Dawley rats)	10, 20, or 40 mg/kg	• Improved motor function and gastrointestinal symptoms • Restored gut microbiota balance by decreasing harmful bacteria (*E. coli*, *Salmonella*) while enriching *Prevotella* that produce short-chain fatty acids • Supports intestinal barrier and BBB integrity • Reduced α-Syn aggregation and protected dopaminergic neurons in the substantia nigra • Suppressed the PI3K/AKT/mTOR signaling pathway, thereby promoting autophagy and enhancing α-Syn clearance	Yu et al. (2024)
Human umbilical-cord stem/progenitor cells (aged wild-type and α-syn transgenic mice)	Intravenous transplantation of human umbilical-cord stem/progenitor cells	• Restored dopaminergic neuronal survival • Alleviated motor impairments • Decreased α-Syn and inflammatory microbiota/cytokines in gut and brain • Intra-gut transplantation increased inflammation, motility, and α-Syn burden, emphasizing the dangers of invasive gut targeting	Lee et al. (2023)
Probiotics (*Bifidobacterium breve* CCFM1067, *Lactobacillus salivarius* AP-32) (6-OHDA male Sprague-Dawley rats)	Oral gavage: 1 mg/mL in distilled water once a day for 8 weeks	• Restored healthy microbial composition • Enhanced SCFA production • Improved intestinal barrier integrity • Reduced neuroinflammation and motor deficits	Tsao et al. (2021)
Probiotic (*Bifidobacterium breve Bif11*) (MPTP female Sprague-Dawley rats)	Oral suspension; 1 × 10^10^ or 2 × 10^10^ CFU daily for 21 days	• Alleviated cognitive and motor impairments • Improved SCFA production and restored intestinal barrier integrity • Preserved tyrosine hydroxylase levels • Reduced oxidative stress and neuroinflammation	Valvaikar et al. (2024)
Er-Bai-Tang decoction (rotenone Sprague-Dawley rats)	EBT decoction, 23.43 g/kg (equivalent crude drug), made at 0.78 g/mL; given via oral gavage, 3 mL each time, twice daily for 30 days	• Downregulated the p38 MAPK pathway in the SN • Restored the composition of the gut microbiota, specifically regulating the Rikenellaceae, Alistipes, and Allobaculum • Enhanced the Sphingolipid metabolism of their intestinal flora	Li et al. (2022)
Fecal Microbiota Transplant (FMT) (MPTP male C57BL/6J mice)	Oral gavage was used to transplant the microbiota, with 0.2 mL per mouse administered once daily for 10 days	• Reduced behavioral deficits • Reduced glial activation • Maintained the integrity of the BBB and increased mitochondrial antioxidative capacity through AMPK/SOD2 • Restored gut dysbacteriosis • Recovering nigrostriatal pericytes and maintaining BBB integrity	Xie et al. (2023)
Fecal Microbiota Transplant (FMT) (Rotenone male C57BL/6J mice)	oral gavage of FMT during weeks 5–6 once per day	• Restored microbiota composition, improving gastrointestinal and motor deficits • Decreased systemic inflammation by reducing gut inflammation and barrier disruption • Reduced LPS levels in the colon, serum, and SN, decreasing LPS-TLR4-induced neuroinflammation • protected dopaminergic neurons against rotenone-induced injury	Zhao et al. (2021)
Panaxadiol (Rotenone male C57BL/6 mice)	25 and 50 mg/kg/day orally for 4 weeks	• Restored healthy microbial communities, decreased taxa linked to inflammation, and balanced the composition of the gut microbiota • Reduced neuroinflammation in the SN and BBB damage was mitigated • Suppressed TLR4/MyD88/NF-κB signaling and downstream inflammatory mediators in the (SN	Xu et al. (2025)
Polymannuronic acid (MPTP male C57BL/6J mice)	(30 mg/kg) daily for 4 weeks by oral gavage	• Modulated gut microbial composition and increased SCFAs • Increased tight junction proteins (such as ZO-1, occludin, and claudins) to improve intestinal and BBB integrity • Reduced inflammation in the brain, stomach, and systemic areas by blocking the MAPK signaling pathway and downregulating proinflammatory cytokines • Improved motor function • Elevated striatal neurotransmitter levels (HVA, 5-HT, 5-HIAA, and GABA)	Dong et al. (2020)

## 6 Discussion

The present review emphasizes mounting evidence suggesting that the PD pathogenesis is linked to gut dysbiosis and BBB disruption. Recent research indicates that peripheral processes involving microbial imbalance, intestinal permeability, and systemic inflammatory signaling significantly contribute to PD progression, alongside dopaminergic degradation in the substantia nigra (Desouky et al., 2024). Experiments consistently show that microbiota alterations enhance neuroinflammation and α-Syn aggregation by promoting disruption of tight junction proteins, systemic inflammation, and BBB dysfunction (Houser and Tansey, 2017; Zhang et al., 2023).

Despite the Diversity of interventions and mechanisms summarized in Table 1, most therapeutic interventions, whether these approaches relied on FMT, probiotics, natural compounds, or stem-cell therapy, a recurring mechanistic pattern observed in these interventions is the modulation of inflammation-related pathways that link gut dysbiosis to central neuroinflammation by modulating gut microbiota, restoring intestinal barrier integrity, enriching SCFAs, and suppressing pro-inflammatory signaling pathways such as PI3K/AKT/mTOR regulation in piperine, which facilitated the autophagic clearance of α-Syn, whereas polymannuronic acid enhanced the expression of tight-junction proteins such as occludin and ZO-1. FMT reduced glial activation and restored mitochondrial antioxidative protection via AMPK/SOD2 signaling. Similarly, Panaxadiol and herbal formulations reduced TLR4/MyD88/NF-κB and MAPK activation. Together, these actions enhance motor outcomes in PD animal models, decrease BBB disruption, and attenuate α-Syn aggregation. Collectively, this highlights the role of the GBA as a central therapeutic target.

Nevertheless, the translational applicability of these findings should be approached with caution since these data derive from toxin-induced PD animal models that include MPTP, rotenone, and 6-OHDA, which imitate specific clinical symptoms but do not fully reflect the chronic and heterogeneous nature of human PD. In addition, diversity in animal models, treatment durations, microbiome assessment methodologies, and outcome evaluation metrics prevents direct comparisons between research. Furthermore, human research keeps demonstrating heterogeneity in microbial diversity across PD patients, most probably due to variations in geographical location, nutrition, illness stage, and medication exposure. This diversity hampers the development of a common microbial signature in PD and may partly explain why microbiota-based therapy options have not yet achieved consistent clinical implementation (Romano et al., 2021; Menozzi and Schapira, 2024).

Another crucial aspect is that commonly used PD symptomatic medications themselves interact bidirectionally with the gut microbiome, potentially affecting barrier-associated pathways. Levodopa, the main PD treatment, is vulnerable to microbial degradation triggered by bacterial tyrosine decarboxylase, limiting its bioavailability preceding intestinal absorption. Moreover, chronic levodopa administration may alter the microbiota composition, possibly exacerbating the dysbiosis already observed in PD patients (van Kessel et al., 2019). While STN-DBS predominantly targets central motor circuits, it may have an indirect effect on the GBA by improving autonomic dysfunction and gastrointestinal transit. Clinical evidence suggests that STN-DBS can alleviate constipation and gastric emptying in some PD patients, presumably altering microbial composition. However, mechanistic evidence tying STN-DBS directly to BBB preservation or microbiota reorganization remains limited, suggesting an essential field for further research (Gorecka-Mazur et al., 2024).

Future research is needed to focus on longitudinal human studies that combine microbiome sequencing, blood-brain barrier biomarkers, and clinical phenotyping in order to evaluate whether microbiota-targeted therapeutics can elicit long-term therapeutic effects. Additional effort should be dedicated to analyzing microbial fingerprints for treatment response prediction, as well as determining if integrated therapy techniques that target microbiota, inflammation, and barrier disruption provide superior clinical benefit.

Collectively, current research reinforces the theory that therapeutics targeting the GBA and restoring intestinal and BBB integrity can complement traditional symptomatic therapies and facilitate PD management. Yet, translation from experimental animal models to clinical application necessitates properly designed clinical studies that can differentiate causal microbiota mechanisms from associative data and determine whether therapies provide clinically meaningful neuroprotective effects.
